# The solid organ transplantation landscape in Chile: a focus on basic and clinical research

**DOI:** 10.3389/fmed.2026.1886870

**Published:** 2026-07-21

**Authors:** Cristián A. Amador, Paola Krall, Elizabeth Galdames, Jorge Morales, Karina Pino-Lagos

**Affiliations:** 1 Facultad de Ciencias, Universidad San Sebastián, Santiago, Chile; 2 Departamento de Pediatría y Cirugía Infantil Oriente, Centro de Investigación Clínica Avanzada (CICA)-Hospital Dr. Luis Calvo Mackenna, Facultad de Medicina, Universidad de Chile, Santiago, Chile; 3 Programa de Trasplante, Clínica Universidad de los Andes, Santiago, Chile; 4 Facultad de Medicina, Centro de Investigación e Innovación Biomédica, Universidad de los Andes, Santiago, Chile

**Keywords:** transplantation in Latin America, transplantation research, funding limitation, challenges in transplantation, Latin America

## Abstract

Transplantation in Chile began after the first global experiences, only 10-years separated the world’s first organ transplant from those initiated in Chile. Currently, Chile performs >600 transplants/year, while 2,000 individuals remain on the waiting list. Advances in Transplantation rely on basic and clinical research, which at the end of the day will impact people’s quality of life and the country’s development as new discoveries and therapies emerge. A clear example of this observation is the link between the excellence and prestige of an academic institution and the country of origin of new discoveries, which at the same time is characterized by a high level of economic resources allocated to science and technology development. Here, we discuss the status of the Chilean Transplantation field, highlighting obstacles and stressing the importance of scientific research for the development of medicine.

## Introduction

**Figure 1:**
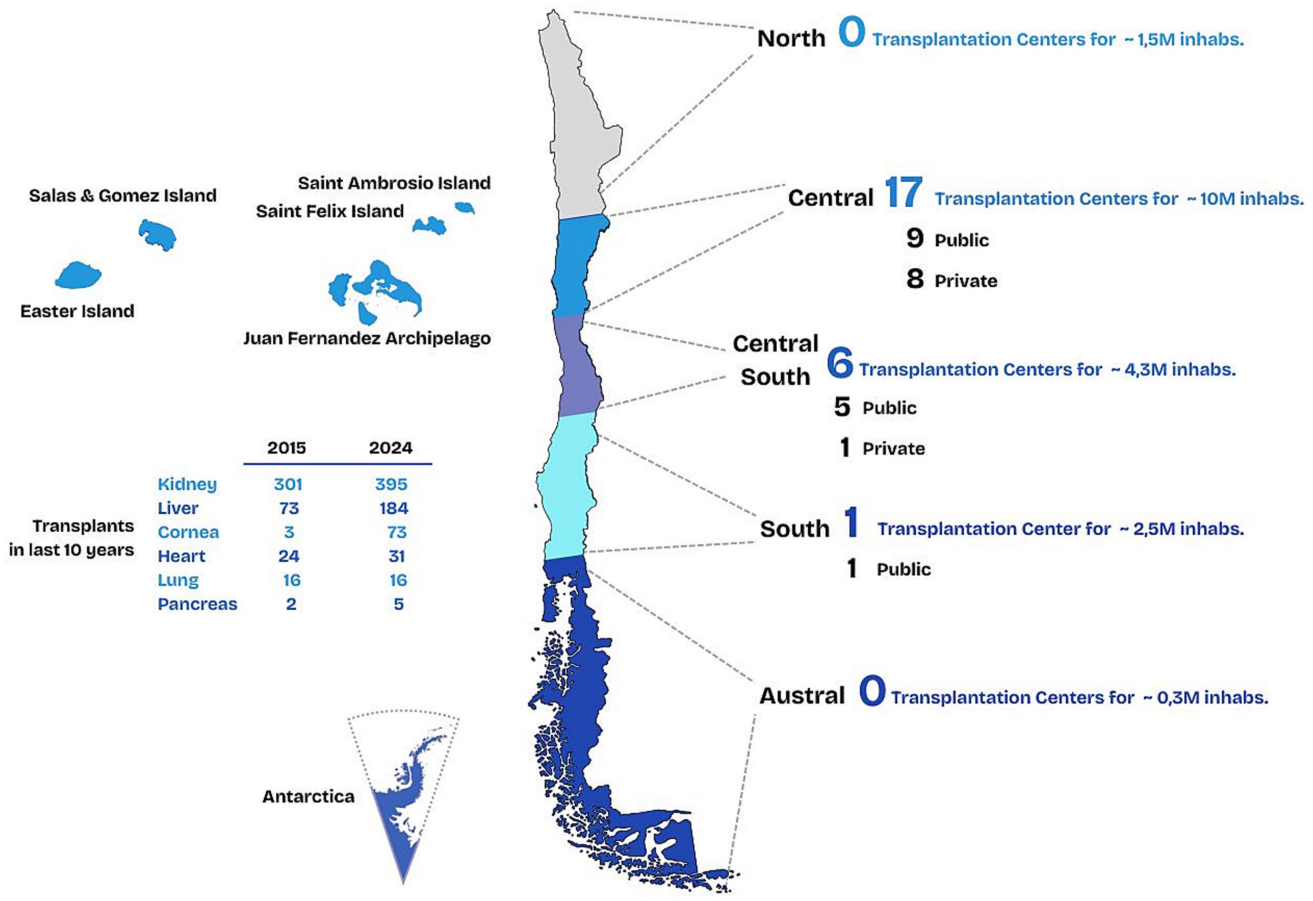
Activity of Organ Transplantation in last decade in Chile. Number of transplants performed between 2015–2024, and approx. Number of inhabitants (inhabs.) per macro-zones. In 2020, the Chilean territory, composed by continental, insular and Antarctic regions, was divided into the North, Central, Central-South, South and Austral macro-zones to decentralize and facilitate the administration of the Ministry of Sciences, Technology, Knowledge and Innovation. Based on this organization, the distribution of public and private Transplantation Centers is shown, highlighting the absence of this service in the extreme zones of Chile and the concentration of them in the Metropolitan Region (located in the Central macro-zone). Image created using elements from Canva.com, licensed under Free Content License.

Chile is one of the world’s longest countries with 4.300 Km in length, including continental, insular and Antarctic territories with about 19 million inhabitants (1). The political organization clusters in Santiago, the country’s capital, which belongs to the Metropolitan Region (MR), concentrating most of the health centers with transplantation units (17 out of 24, with 9 public and 8 private ones). The North and Austral macro-zones of the country have no transplantation units, whereas the Central-South and South macro-zones group 7 institutions in total (6 public and 1 private) (2) (Figure 1). With this structure, Chile reported from 2015 to 2024: 1,572 effective donors resulting in 6,040 transplants, with 91% originating from public health institutions, 80% corresponding to deceased donors and 20% to living donors and presenting a mean donor age of 45 years (61% male). Seventy percent of donors came from the Central-South macro-zones. In 2015 the top 3 transplanted organs corresponded to kidney (n = 301), liver (n = 73) and heart (n = 24). However, in 2024 cornea overpassed heart transplantation, reaching 73 procedures, and kidney and liver transplantation increased to 395 and 184, respectively. Smaller increments were observed for heart, lung and pancreas, reaching 31, 16 and 5 procedures in 2024, respectively (3) (Figure 1). This scenario is supported by the national health system, which is composed of a public and private sector. The public sector covers 80% of the population, representing close to 50% of the total health expenditure, whereas the other 50% is covered by “Instituciones de Salud Previsional” (ISAPRES) or private health insurances, and out-of-pocket payments (4). Despite these numbers, we sought to investigate whether (and how) this transplantation activity is being supported by basic and clinical research: how many research groups exist in the country (and their location)? Is publication accompanying investigation in transplantation? How active is the transplantation community (researchers, clinicians) in Chile? To answer these questions, we consulted the web, including databases from Chilean scientific and medical societies, and Advanced Search in Pubmed. We found that only the Chilean Society of Transplantation and the Chilean Society of Nephrology received research abstracts for their corresponding national meetings, with 201 studies presented between 2015–2025. Of them, 8% correspond to basic research performed by only 2 academic-basic research laboratories placed in 2 private universities located in Santiago. The remaining studies (> 90%) covered a wide array of topics from clinical reports, post-transplant follow-ups, centers experiences, reports on surgical techniques, and studies including legal aspects, patients’ perception of waiting lists, and coordination of intensive care personnel. Given that basic research forms the foundation of applied and translational disciplines, the current state of transplantation-related basic research in Chile is concerning. In other words, how could we contribute -from Chile- to the advances of the transplantation field if research is limited? Regarding scientific publications, we used 2 search criteria: “Organ Transplantation” and “Transplantation Tolerance” as keywords, refining with “Chile” for affiliation, and “last decade” for date. Manually, we then selected those in which the corresponding author belongs to a Chilean institution. With the first keywords we found 120 publications, most of them case reports and reviews. For the second criteria, we only found 8 papers, mainly on basic research. Just considering the existing Transplantation centers, Chile has an annual rate of 0.53 publication/center in the Transplantation field.

## Moving forward

To date, Chile has contributed to Transplantation research driven by universities and by public and private health centers. The current funding system is recognized as one of the main limitations for the growth of this discipline, together with the geographic distribution of the Transplantation units along the country. In 1967, the “Consejo Nacional para la Investigación y Tecnología” (CONICYT) was created, an entity that by 2017 had 13 programs to fund investigation, distributing the national budget (0,3-0,4% of Gross Domestic Product (5)) among all disciplines (humanities, natural sciences, including biomedical ones). Of these programs, the “Fondo Nacional para la Investigación y Tecnología” (FONDECYT) offered 3 types of grants with only one call suitable to fund basic and clinical research, with a total budget of approx. USD$200,000 for a 4-year grant (6). In 2020, CONICYT changed its name to “Agencia Nacional de Investigación y Desarrollo” (ANID), with the promise to support the national research in responding to the current demands of the society and placing Chile as a country where science of excellence is executed. For instance, ANID created new grant calls directed to finance research centers, international collaboration and industry. Regarding biomedical investigation, the calls usually establish -in advance by ministerial board of advisors- defined health topics (for example, cancer, mental health, well-being of the elderly, obesity, among others), with transplantation not receiving equal interest as no explicit call has intended to foster this area of medicine (7). From another perspective, clinical research infrastructure (biobanks, data platforms, and trained personnel) needs to be developed and decentralized. Emerging opportunities could strengthen research transplantation, including the growing availability of anonymized national registries, advances in personalized medicine, and new collaborations. This requires coordination among stakeholders - government, universities, health services, and scientific/medical societies-, along with policies promoting research in transplantation.

## Conclusion

We summarized the current state of transplantation in Chile, discussing obstacles and future directions to foster adequate growth that meets societal needs. Although not mentioned above, Chilean society is built by diverse cultures and ethnicities, both endemic and foreign ones, offering a degree of complexity to Transplantation Research that could be of interest to the international scientific community. We urge the promotion and support of basic/clinical research, as strengthening the knowledge base will be key to positioning Chile and Latin America in line with global transplantation standards.

## Data Availability

The original contributions presented in the study are included in the article/supplementary material, further inquiries can be directed to the corresponding author.
